# In Vitro Biological Evaluation of an Alginate-Based Hydrogel Loaded with Rifampicin for Wound Care

**DOI:** 10.3390/ph17070943

**Published:** 2024-07-14

**Authors:** Tudor Bibire, Radu Dănilă, Cătălina Natalia Yilmaz, Liliana Verestiuc, Isabella Nacu, Ramona Gabriela Ursu, Cristina Mihaela Ghiciuc

**Affiliations:** 1 Doctoral School, Grigore T. Popa University of Medicine and Pharmacy, 16 Universitatii Street, 700116 Iasi, Romania; tudor_cd_bibire@d.umfiasi.ro; 2 St. Spiridon County Clinical Emergency Hospital, 1 Independentei Blvd., 700111 Iasi, Romania; radu.danila@umfiasi.ro; 3 Department of Surgery, Faculty of Medicine, Grigore T. Popa University of Medicine and Pharmacy, 16 Universitatii Street, 700116 Iasi, Romania; 4 Biochemistry Division, Department of Chemistry, Faculty of Science, Dokuz Eylül University, Kültür Mah. Cumhuriyet Bulv. No:144 Alsancak, 35210 Izmir, Turkey; 5 Department of Biomedical Sciences, Faculty of Medical Bioengineering, Grigore T. Popa University of Medicine and Pharmacy, 16 Universitatii Street, 700116 Iasi, Romania; cobzariu.isabella@gmail.com; 6 Petru Poni Institute of Macromolecular Chemistry, 41-A Grigore Ghica Voda Alley, 700487 Iasi, Romania; 7 Department of Microbiology, Faculty of Medicine, Grigore T. Popa University of Medicine and Pharmacy, 16 Universitatii Street, 700116 Iasi, Romania; ramona.ursu@umfiasi.ro; 8 Department of Pharmacology, Faculty of Medicine, Clinical Pharmacology and Algeziology, Grigore T. Popa University of Medicine and Pharmacy, 16 Universitatii Street, 700116 Iasi, Romania; cristina.ghiciuc@umfiasi.ro; 9 St. Maria Clinical Emergency Hospital for Children, 62 Vasile Lupu Street, 700309 Iasi, Romania

**Keywords:** wound care, hydrogel, antimicrobial, bioadhesion, wound healing

## Abstract

We report a biocompatible hydrogel dressing based on sodium alginate-grafted poly(N-vinylcaprolactam) prepared by encapsulation of Rifampicin as an antimicrobial drug and stabilizing the matrix through the repeated freeze–thawing method. The hydrogel structure and polymer-drug compatibility were confirmed by FTIR, and a series of hydrogen-bond-based interactions between alginate and Rifampicin were identified. A concentration of 0.69% Rifampicin was found in the polymeric matrix using HPLC analysis and spectrophotometric UV–Vis methods. The hydrogel’s morphology was evaluated by scanning electron microscopy, and various sizes and shapes of pores, ranging from almost spherical geometries to irregular ones, with a smooth surface of the pore walls and high interconnectivity in the presence of the drug, were identified. The hydrogels are bioadhesive, and the adhesion strength increased after Rifampicin was encapsulated into the polymeric matrix, which suggests that these compositions are suitable for wound dressings. Antimicrobial activity against *S. aureus* and MRSA, with an increased effect in the presence of the drug, was also found in the newly prepared hydrogels. In vitro biological evaluation demonstrated the cytocompatibility of the hydrogels and their ability to stimulate cell multiplication and mutual cell communication. The in vitro scratch assay demonstrated the drug-loaded alginate-grafted poly(N-vinylcaprolactam) hydrogel’s ability to stimulate cell migration and wound closure. All of these results suggest that the prepared hydrogels can be used as antimicrobial materials for wound healing and care applications.

## 1. Introduction

Modern wound dressings containing hydrogels with antibacterial properties can contribute essentially to the appropriate care of wounds (surgical wounds, trauma, or accident wounds) by promoting and accelerating the wound healing process and reducing the risk of complications and infection that could turn them into chronic wounds. Because of the growing resistance of bacteria to antibiotics and the formation of microbial biofilm, which enhances the risk of wound infection and is one of the most important barriers in the wound healing process, particularly for chronic wounds, wounds represent a major source of expenses. Surgical wounds are a significant source of costs for the healthcare system, particularly when it comes to treating infected wounds, which is seen in the number of hospital days, the number of care days for patients, and the days that patients take to return to normal activity in society. Thus, surgical wounds represent some of the most substantial expenses, especially when considering the care of infected wounds [1,2,3,4,5,6]. Due to their similarity to the extracellular matrix and compatibility with the biological environment that is absolutely necessary for biomedical applications, biopolymers from natural sources, such as alginic acid and alginates, are suitable to be formulated as suitable hydrogels for wound care of various etiologies, including postoperative wounds.

Alginic acid and its derivatives have been shown in numerous studies to have their own biological properties, including antimicrobial and antifungal activity, anti-inflammatory effects and regenerative and angiogenetic properties, immunomodulatory and antioxidant effects, as well as hemostatic action [6,7,8,9]. Alginic acid, a polysaccharide extracted from brown seaweed, has and possesses outstanding physical characteristics that make it valuable for medical and pharmaceutical applications. It was decided to choose alginic acid to obtain and study hydrogels because of its important properties, namely viscosity and ability to form gels. Alginates are copolymers formed from repeating units of guluronic acid (G) and β-D-mannuronic acid (M), linked by 1,4-glycosidic bonds. They are each other’s C5 epimers in terms of molecules. The orientation of the carboxyl group (–COOH) on the C5 carbon of the six-membered saccharide ring is above the plane of the ring in the M unit and below the plane in the G unit. The polymer chain of alginate contains blocks of guluronic acid, mannuronic acid, and alternating sequences of both units. In addition, with proper functionalization, their poor mechanical properties and dependence on various environmental factors can be significantly improved [9,10].

Therefore, the combination of alginic acid with biocompatible monomers, such as N-vinylcaprolactam (NVCL), can lead to functional biocompatible materials that preserve both alginate and NVCL characteristics. Graft copolymerization of plant polysaccharides offers the advantage of the introduction of functional groups (e.g., thermoresponsive or pH-responsive moieties) onto the polysaccharide backbone to improve intrinsic properties like rheological properties, hydrophilicity, polymer charges, molecular chains’ aggregation, and complexing capability contributing to the final outcome of the materials [11].

Poly(N-vinylcaprolactam) (PNVCL) is part of the N-vinylamide polymer class and is a water-soluble, non-toxic, thermosensitive, and biocompatible polymer. The polymer exhibits a lower critical solution temperature (LCST) that is comparable to physiological conditions (32–34 °C), as does poly(N-isopropylacrylamide) (PNIPAAm). However, we consider that PNVCL shows higher biocompatibility because the amide group is directly linked to the hydrophobic main chain (C–C) and, as a result, the hydrolysis of the amide group in PNVCL does not provide small amide compounds, which would be undesirable for our intended purpose [12,13,14,15].

The prevention of wound infection or more effective treatment of infected wounds frequently in patients with pre-existing pathologies (diabetes, venous disease, cardiovascular disease, obesity, immunosuppressed patients) requires the addition of antimicrobial molecules [16,17] to prevent the wound from becoming chronic and to allow the healing process. Such interventions lead to a reduction in the great economic effort and serious problems for health systems worldwide [18,19]. Rifampicin (Rif) or rifampin is a hydrophobic antibiotic of the rifamycin class and a major anti-tuberculosis agent in the first-line treatment of *Mycobacterium tuberculosis*-induced tuberculosis, along with other anti-tuberculosis agents. Moreover, it has antibacterial activity against a broad spectrum of Gram-positive and Gram-negative microorganisms (*Neisseria meningitidis*, *Neisseria gonorrhoeae*, *Clostridium difficile*, *Haemophilus influenzae*, *Staphylococcus aureus*, methicillin-resistant *Staphylococcus aureus* (MRSA), and *Staphylococcus epidermidis*) [20,21,22,23,24,25,26,27,28,29,30], which supports investigating the drug for wound care and healing, especially infected or susceptible wounds, as this can contribute to the improvement in patients’ quality of life, also by increasing their treatment adherence [31,32]. Moreover, Wallenwein et al. reported that Rif has anti-inflammatory properties that accelerate the healing process of chronic wounds; it is well known that high levels of proinflammatory cytokines in chronic wounds prolong the wound-healing process [33].

This study aimed to evaluate the in vitro biological activity (cytotoxicity, wound healing effect) and antimicrobial activity of a newly developed hydrogel based on alginate grafted with poly(N-vinylcaprolactam) (PNVCL) and loaded with Rifampicin.

## 2. Results and Discussions

### 2.1. Hydrogel Preparation and Structure

**Figure 1:**
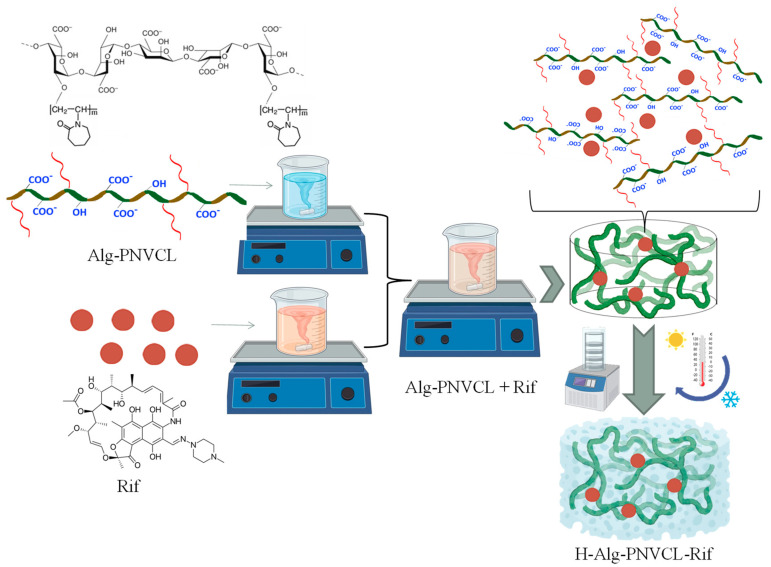
Description of the preparation steps of the hydrogel based on sodium alginate grafted with poly(N-vinylcaprolactam) and loaded with Rifampicin via solution mixing and freeze–thawing processes.

Newly synthesized copolymers based on alginate and PNVCL and thermosensitive and biocompatible materials were formulated as hydrogels by loading Rifampicin (Rif) as a model drug using non-invasive and efficient physical crosslinking methods during repeated freeze–thawing processes (Figure 1).

PNVCL grafted on sodium alginate is a copolymer with thermoresponsive characteristics [34,35], which can be exploited in drug delivery to protect the drug against degradation and fast release in the human body. The drug loading (i.e., Rifampicin Rif) was performed by mixing solutions of Rifampicin and the copolymer (concentration of 2 wt%). The theoretical amount of Rif used for loading was calculated to be 0.5 wt% of the polymeric matrix. The mixing was left overnight to homogenize well, and the resulting solution was freeze-dried and further processed as hydrogels. The mixture was easily processed as a porous hydrogel by the freeze–thawing method. During repeated freezing and thawing, the polymer and drug interact, and the molecules rearrange and stabilize the structure. Rifampicin forms hydrogen bonds with hydrophilic groups from alginate, and PNVCL interacts via a combination of hydrophobic/hydrophilic interactions [36], which are strongly dependent on temperature.

**Figure 2:**
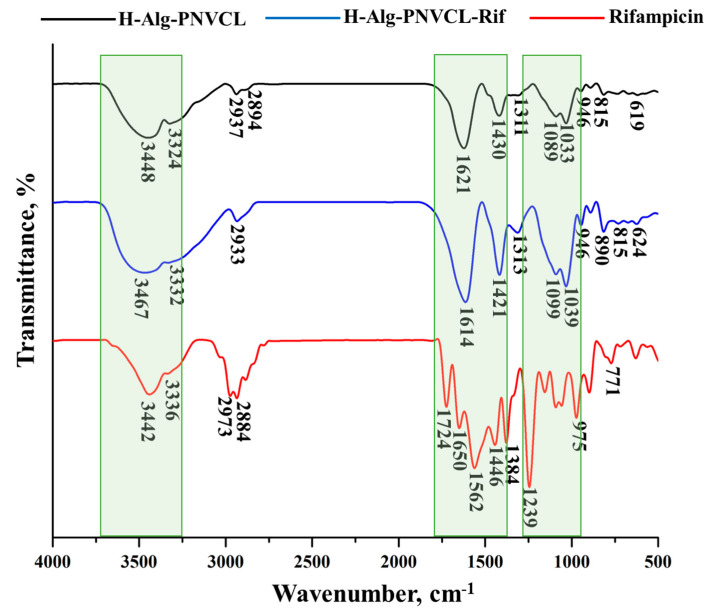
FTIR spectra: H -Alg-PNVCL, H-Alg-PNVCL-Rif, and Rifampicin.

The hydrogel’s structure was investigated by FT-IR spectroscopy, and the resulting spectra are presented in Figure 2.

The peak observed on FT-IR spectra of H-Alg-PNVCL at 3448 cm^−1^ corresponds to the stretching vibrations of hydroxyl groups. An intense absorption band was registered at 1621 cm^−1^, attributed to the characteristic absorption of carbonyl groups in the amide ring structure (N, N′-dialkyl amide) of the grafted copolymer [34]. The peaks from 2937 cm^−1^ and 2894 cm^−1^ are attributed to the stretching of aliphatic C–H bonds. The C–N stretching vibration reached its highest point at 1430 cm^−1^, whereas the N–H stretching vibrations were identified at 3332 cm^−1^ [34]. On the other hand, in the bioactive compound’s spectrum, Rifampicin [31] presents some characteristic absorptions: 3442 cm^−1^ (NH stretching), 2973 cm^−1^ (C-H bonding), 1724 (C=O, acetyl stretching), 1650 (C=N, asymmetric stretching), 1562 cm^−1^ (NH, amide bending), 1446 cm^−1^ (C=C), 1384 cm^−1^ (CH_2_, C=C), 1239 cm^−1^ (–CH, CO, C–H), and 975 cm^−1^ (C≡C-H, C–H) [32]. In the H-Alg-PNVCL-Rif spectrum, the absorption attributed to C–N stretching is intensified, as well as the absorptions from 1039 cm^−1^ and 1089 cm^−1^, demonstrating the hydrogen bonds between the Alg-PNVCL copolymer and the bioactive compound. The peak seen at 2933 cm^−1^ suggests the elongation of aliphatic C–H bonds, and the intensification of the N–H and O–H stretching vibrations were noticed at 3332 cm^−1^ and 3447 cm^−1^, respectively, indicating that the polymeric system was formed, and Rifampicin was successfully encapsulated in the polymeric matrix.

### 2.2. SEM Data—Hydrogel Morphology

**Figure 3:**
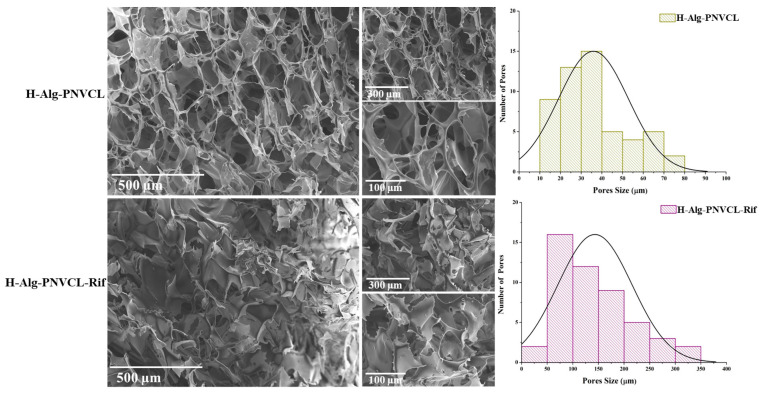
SEM data and porosity distribution of H-Alg-PNVCL and H-Alg-PNVCL-Rif hydrogels.

The H-Alg-PNVCL and H-Alg-PNVCL-Rif hydrogels present a porous structure with different pore sizes and pore shapes due to the syneresis processes produced during the freeze–thawing steps and polymeric matrix–drug interactions. The H-Alg-PNVCL hydrogel presents a uniform distribution of the pores and almost spherical geometry, with a smooth pore surface and high interconnectivity, as can be observed at high magnification in Figure 3. The ImageJ software v1.51 was used to calculate the pore sizes and pore size distribution [37]. The drug inclusion changes the copolymer distribution, and the 3D hydrogel network presents a non-uniform distribution and irregular shape. The loading of Rifampicin inside the hydrogel matrix can induce a non-uniform expanding effect on the polymeric matrix (hydrophilic backbone) during the repeated freeze–thawing steps and the pore size increase. For instance, in Figure 3, the H-Alg-PNVCL hydrogel has pores varying from 10 µm to 80 µm (Table 1), while in the H-Alg-PNVCL-Rif hydrogel, the pore size ranges from 10 µm to 340 µm.

Under testing conditions, the hydrogel samples were slowly frozen at −20 °C, which generally led to large ice crystals [38,39]. Furthermore, while the crystals are growing, the gel expands, which potentially damages the gel structure [40]. During the drying step, the sublimation of the ice crystals leaves behind a homogenous/non-homogenous porous network and can produce shrinkage of the hydrogel matrix [41]. Polymer structure, molecular weight, the concentration of the solution, and some additives can regulate the final structure of the porous network. The drug dissolution into the polymeric matrix influences the freeze-drying process by drug distribution around hydrophilic groups from alginate, and the resulting structure is non-uniform with collapsed parts. PNVCL, as a temperature-responsive branch [42], forms inter-chain bonds. These results indicated that PNVCL could be used as an effective filler to reinforce Alg-based wound dressing materials containing a hydrophobic drug. Morphological parameters, including total porosity, pore geometry, mean pore size, and pore interconnectivity, are crucial factors that define their applicability as wound dressings.

Hydrogels with higher interconnectivity and uniform porosity are recommended for cellular growth, as a large surface is available for cell adhesion and migration.

### 2.3. Drug-Loading Capacity and Release

**Figure 4:**
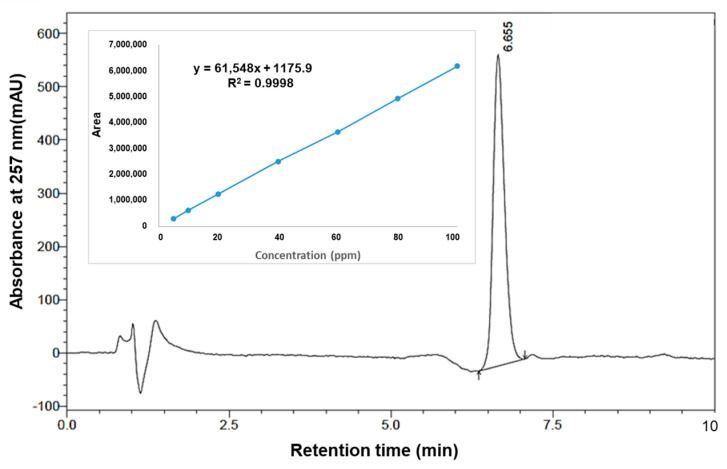
HPLC chromatogram of the Rifampicin-loaded hydrogel and the calibration curve for drug release.

The amount of Rifampicin loaded in the hydrogel was calculated based on a developed HPLC method and a spectrophotometric method, respectively. The HPLC chromatogram of the Rifampicin-loaded hydrogel and the calibration curve for drug release are presented in Figure 4.

In each assay method, degrees of Rifampicin loading in the hydrogel were 0.69% Rif and 0.68% Rif, respectively. The drug release results revealed that the equilibrium of diffusion was reached after 4 h, and the loaded drug was transferred to the testing medium. The amount of released Rif was very low (5.98 ppm), and we consider the outcome to be due to the small concentrations of Rif loaded in hydrogel and the testing conditions.

### 2.4. Bioadhesive Properties

Bioadhesive polymers (e.g., proteins and polysaccharides) adhere to specific chemical structures in tissue (glycoproteins, cysteine-rich subdomains) and cells (receptors and cell adhesion ligands) and regulate the cell–material and tissue–material relations [43,44]. Bioadhesive polymers, whose main function is the sealing of the lesion, work based on a balance between adhesion to human tissues and cohesion forces to sustain their maneuverability [45]. However, in traditional polymers, these two characteristics are often in conflict, and the modulation of the properties can be achieved by modifying side groups for balancing covalent or non-covalent interactions, crosslinking, or polymer grafting [46]. The collapse or relaxation of side chains into grafted copolymers controls the behavior in the biological environment, binds to glycoprotein chains or cell membranes, and therefore contributes to the bioadhesion of the dressing material [47,48].

**Figure 5:**
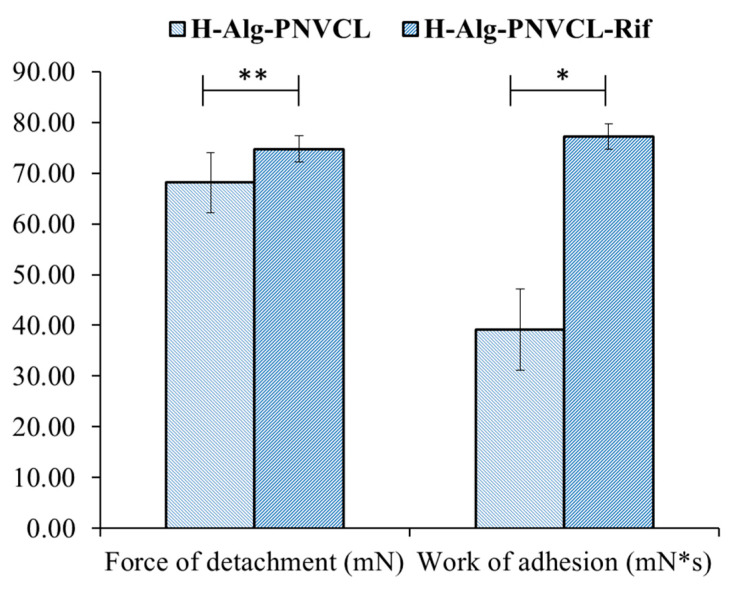
Detachment force and the work of adhesion for H-Alg-PNVCL and H-Alg-PNVCL-Rif hydrogels (* *p* < 0.05, ** *p* < 0.01).

Various in vitro testing membranes/ex vivo tissues/in vivo procedures have been used to evaluate the material’s bioadhesive properties, and good correlations between methods have been observed when physiological conditions are correctly simulated [49]. In vitro bioadhesion studies of the H-Alg-PNVCL and H-Alg-PNVCL-Rif hydrogels were performed on a TA.XT Plus texture analyzer using a cellulose membrane as a simulating medium. The hydrogels and the membrane were maintained in contact, and the force required for detachment of the scaffolds from the membrane and the work of adhesion were calculated (Figure 5).

An increment of 48.25% in bioadhesiveness was registered when Rif was encapsulated in the hydrogel. Alginate is hydrophilic and stimulates hydrogel wettability. PNVCL has a hydrophobic carbon–carbon main chain and hydrophilic cyclic amide side groups that physically interact through diffusion and physical entanglement, simulating biological membranes and contributing to a weak bioadhesive force. The interpenetration of polymer chains, as well as the wettability of the entire structure, the drug content, and drug–matrix interactions, are controllable parameters that can design the bioadhesive characteristics.

### 2.5. Antimicrobial Properties

In this study, two Gram-positive bacterial strains (*S. aureus* and MRSA) and two Gram-negative (*E. coli* and *P. aeruginosa*) were selected for antimicrobial tests. The cell wall for Gram-negative differs from Gram-positive bacteria in terms of LPS (lipopolysaccharide presence) versus thick peptidoglycan, teichoic, and lipoteichoic acids. Different cell wall content reacts differently to the same antibacterial substances. The Rifampicin-loaded hydrogel (H-Alg-PNVCL-Rif) shows very good antimicrobial activity against *S. aureus* (26/28 mm diameters inhibition areas) and MRSA (24/26 mm) (Table 2). The unloaded hydrogel (H-Alg-PNVCL) also has good antimicrobial action against *S. aureus* and MRSA, which supports this type of matrix for in vivo studies for potential use in therapy, especially after functionalization with antimicrobial substances, such as Rifampicin, in which case the antimicrobial efficacy of the hydrogel with Rifampicin will significantly increase against the two mentioned microbial strains. Rifampicin pure substance has very good antimicrobial action against all four microbial strains tested, but the toxic effects of high concentrations of Rifampicin are not to be neglected and, therefore, the Rifampicin-loaded hydrogel (H-Alg-PNVCL-Rif) is a preferable alternative in wound care, including postoperative wounds.

In all cases, the inhibition area increased slightly (2 mm) after 48 h, which indicates maintenance of the antimicrobial activity for a long time; hence, the application of a Rifampicin-loaded hydrogel (H-Alg-PNVCL-Rif) in a pharmaceutical form (in the formulation of ointment, dressing, patch, etc.) to the wound can be performed at a fairly long interval and will thus improve patient compliance to proper and individualized wound care.

The scientific literature reports a high prevalence of infected wounds with similar strains as we tested [50,51,52], which emphasizes the importance of developing new therapeutic strategies. Our results are promising, but in order to be implemented in clinical practice, we should validate our developed therapeutic drugs using the rules of *The European Committee on Antimicrobial Susceptibility Testing (EUCAST)* guideline [53].

### 2.6. Hydrogels Cytocompatibility

**Figure 6:**
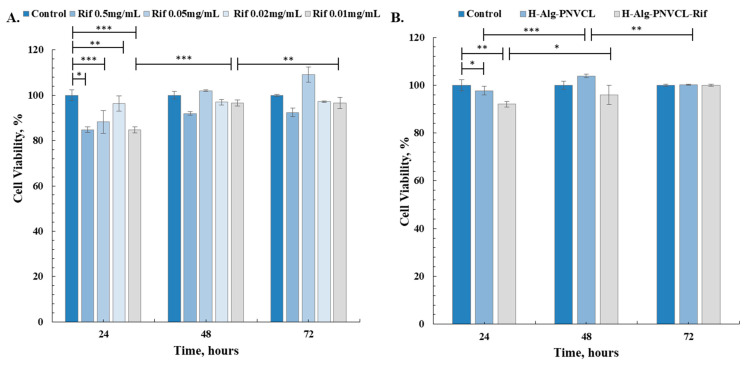
MTT cell viability of NHDF cells for (**A**) Rifampicin solutions and (**B**) H-Alg-PNVCL and H-Alg-PNVCL-Rif hydrogels (n = 3; * *p* < 0.05, ** *p* < 0.01, *** *p* < 0.001).

In order to assess the cytotoxic effects of H-Alg-PNVPM and H-Alg-PNVPM-Rif scaffolds on human dermal fibroblasts (NHDF cell line), an in vitro MTT test was employed, as presented in Figure 6. The MTT test is a widely used technique for investigating biological processes and assessing cell viability by comparing experimental cultures to control ones [54].

An insignificant decrease in cell viability values (measured by mitochondrial dehydrogenase activity) was registered after cells were exposed to the H-Alg-PNVPM hydrogel matrix. Likewise, the value of cell viability for cells in contact with the bioactive compound, Rifampicin, obtained by the extract method (four solutions of 0.5 mg/mL, 0.05 mg/mL, 0.02 mg/mL, and 0.01 mg/mL concentrations) was approximately 98% after 72 h of the experiment. The mitochondrial dehydrogenase activity decreased below 10% for the 0.5 mg/mL of Rifampicin solution and is a level described as non-cytotoxic according to ISO 10993-5 standard [55].

On the other hand, the viability of the cells exposed to the matrices loaded with the bioactive compound was over 95%, compared to the control wells, demonstrating the ability of the matrices to support cell proliferation.

**Figure 7:**
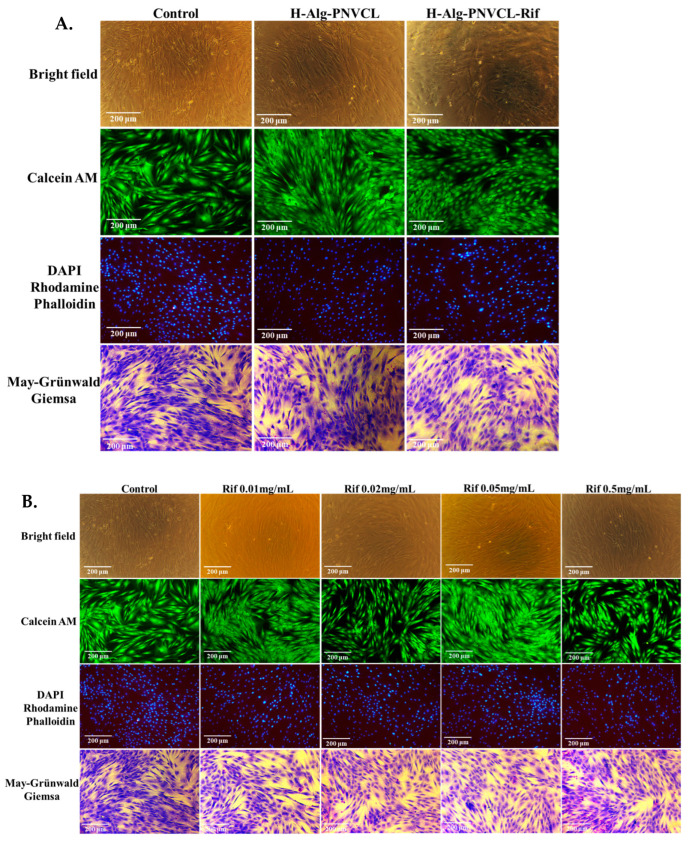
Viable NHDF cells and fixed cells, respectively, after 72 h of cell culturing with H-Alg-PNVCl and H-Alg-PNVCL-Rif hydrogels (**A**) and Rifampicin (**B**).

Normal human dermal fibroblasts (NHDF cell line) were subjected to hydrogels for an average of 72 h. After that, cells exposed to H-Alg-PNVPM, H-Alg-PNVPM-Rif, and Rifampicin solutions with different concentrations were visually analyzed using Giemsa staining, DAPI-Rhodamine Phalloidin, and Calcein-AM staining techniques (as shown in Figure 7). The pictures, taken with a x10 lens, clearly show the presence of the viable cells. The cells exhibit morphological integrity, adhering to the substrate, presenting a consistent monolayer, and displaying the typical shape of healthy fibroblasts.

In addition, the cell culture exhibited a fast proliferation, characterized by fibroblasts displaying spindle morphology. During the subsequent examination, it was seen that the cells underwent rapid multiplication and engaged in mutual communication, strong evidence that the hydrogels developed in this study are suitable for the wound healing process.

### 2.7. Wound Healing Scratch Assay

Wound healing is a complex process and involves several particular phases, such as hemostasis, inflammation, cell proliferation, and tissue remodeling, which occur thanks to the activity of the cells in combination with collagen and other constitutive polymers of the ECM. The growth factors, interleukins, chemokines, and cytokines contribute to a series of molecular and cellular events, stimulate the interactions between cells and cells with components in the extracellular matrix (ECM), heal the wound, and repair the tissue [56].

**Figure 8:**
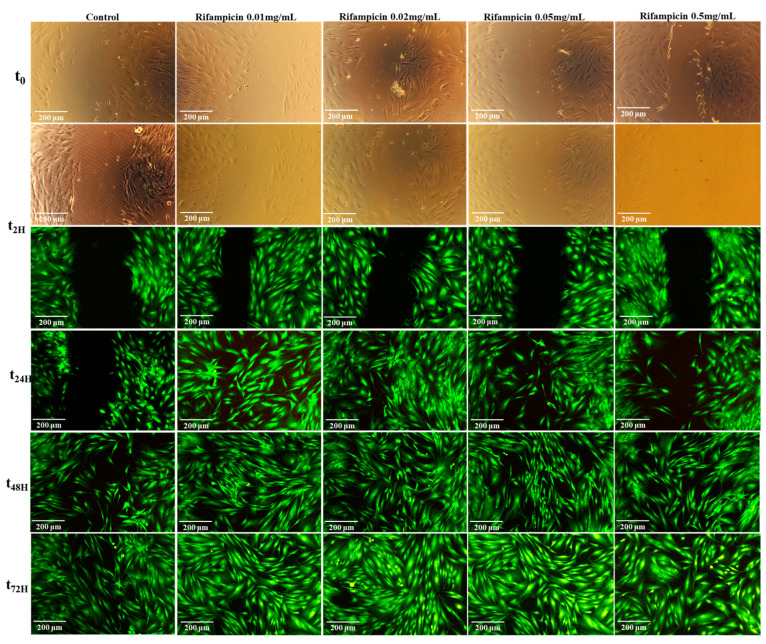
In vitro evaluation of NHDF migration in the presence of different concentrations of Rifampicin (NHDF cell line stained with Calcein AM at various times).

**Figure 9:**
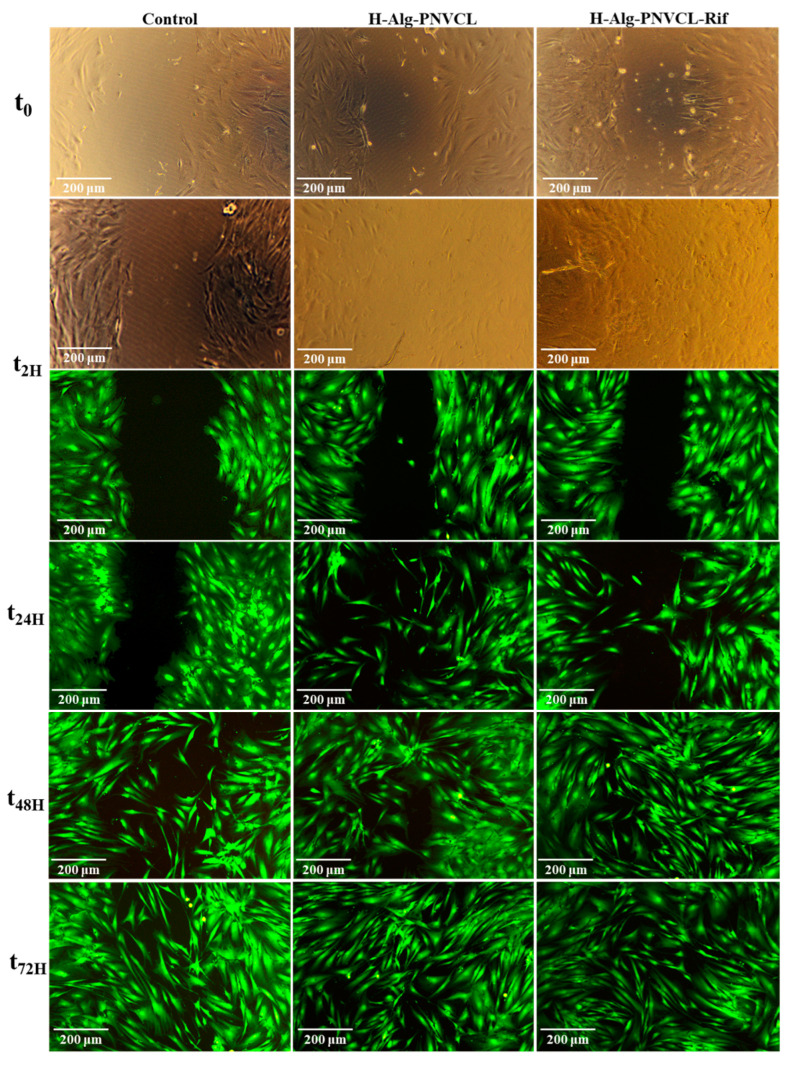
In vitro evaluation of NHDF migration in the presence of H-Alg-PNVCL hydrogel and H-Alg-PNVCL-Rif hydrogel (NHDF cell line stained with Calcein AM at various times).

Various protocols have been developed to evaluate a material’s potential to repair biological tissues, from in vitro wound scratch tests to in vivo studies [57,58]. In our study, the scratch assay was performed to investigate in vitro wound healing process. Migration and proliferation of NHDF cells, a time-dependent (0–72 h) wound scratch assay was conducted for bare hydrogel and drug-loaded hydrogels, as well as with various concentrations of Rifampicin; the material’s effect on wound healing was analyzed by visualization of the wound areas (staining with calcein AM) at different time points (Figure 8 and Figure 9 and Supplementary Materials). The Rifampicin proved to induce an effect of healing at the tested concentrations; after 24 h of the experiment, the wound repair progressed until closure for the concentrations of 0.01 mg/mL and 0.02 mg/mL (Figure 8).

At higher concentrations, the wound healing capacities were detected after 72 h. Rifampicin exhibits activity on anti-Gram-positive bacteria and acts on intracellular bacteria. Some tests evaluated the toxicity of Rifampicin and the drug’s effects on cells and demonstrated no effect on osteoblasts when its concentration did not exceed 10 μg/mL [59] or human bone-marrow-derived MSCs at concentrations above 32 μg/mL [60]. In our testing conditions and concentrations, the Rifampicin presented a regenerative effect.

For the control group (cells with no material), the process of cell migration started at 24 h, and a slow repair was observed even at 72 h. However, there is obvious wound closure for the H-Alg-PNVPM hydrogel and the H-Alg-PNVPM-Rif hydrogel at 48 h. Both hydrogels enhanced the rate of wound closure significantly, starting at 2 h and progressing until complete closure (Figure 9 and Supplementary Materials).

Furthermore, in a scratch model, the Rifampicin-loaded hydrogels demonstrated a synergetic effect between polymeric matrix and drug; the combined hydrogel can stimulate cell proliferation and migration and induce faster wound closure. Alginates have been extensively tested as dressings (hydrogels, nanofibers, films, foams, or topical formulations) due to polymer biocompatibility and hydrophilicity, and some combinations demonstrated the ability to absorb excessive wound fluid, gelling properties, and antibacterial properties [61]. The wound dressing’s efficacy is influenced by the ratio of other small molecules or polymers, the crosslinking processes, and the resulting construct morphology. In our study, the alginate grafting with poly(N-vinylcaprolactam) was favorable to the wound-healing properties of a porous formulated material.

This study has potential limitations associated with the content of Rifampicin in the hydrogel due to Rifampicin toxicity. However, further investigations will be performed to evaluate the maximum safe concentration of the drug encapsulated in modified-release polymeric hydrogel to increase and extend the antimicrobial activity to other bacterial strains and subsequently ensure optimal therapeutic efficacy based on the antimicrobial and anti-inflammatory activity of Rifampicin, as well as the self-healing ability of both Rifampicin and the biopolymeric and thermosensitive matrix.

## 3. Materials and Methods

### 3.1. Materials

Alginic acid (AlgA), sodium salt from brown seaweed, was used as a commercial product obtained from Sigma Aldrich, Hamburg, Germany (CAS 9005-38-3) with a viscosity between 15 and 25 cP for the 1% solutions. As a biocompatible monomer, N-vinylcaprolactam (NVCL) CAS 2235-00-9 was used for grafting on the alginate macrochain, which was obtained from Sigma-Aldrich, Hamburg, Germany. The initiating system (ammonium persulphate—APS, hydrogen peroxide—50% solution) was purchased from Sigma Aldrich, Hamburg, Germany. Rifampicin (Rif, 99% purity, molecular weight of 822.95 g/mol) originated from S.C. Antibiotice S.A. Iasi, Romania, and was used as received. All other chemicals and reagents met analytical-grade standards and were used as received in solutions of twice-distilled water.

All reagents involved in cytotoxicity tests and wound healing assays were purchased from Sigma-Aldrich (Hamburg, Germany) and used as received. For culturing, Dulbecco’s modified Eagle’s medium (DMEM, with 4500 mg/mL glucose, 110 mg/L sodium pyruvate, and 0.584 mg/L l-glutamine) and fetal bovine serum (FBS, sterile-filtered, suitable for cell culture) were used, with penicillin/streptomycin/neomycin solution (P/S/N, with 5000 units penicillin, 5 mg streptomycin, and 10 mg neomycin/mL, sterile-filtered, suitable for cell culture), ethylenediaminetetraacetic acid (EDTA), trypsin, phosphate-buffered saline solution (PBS, sterilized, suitable for cell culture) and 3-(4,5-dimethyl-2-thiazolyl)-2,5-diphenyl-2H-tetrazolium bromide (MTT), and calcein AM.

### 3.2. Hydrogel Preparation

For hydrogel preparation, sodium alginate was grafted with poly(N-vinylcaprolactam) (PNVCL), which was synthesized via the radical polymerization technique of NVCL and H-bonding to alginate COOH groups. The initiating system comprised ammonium persulphate (APS, Sigma Aldrich, Hamburg, Germany) and 50% hydrogen peroxide solution under nitrogen flow within a ratio of 0.1% against the monomer (NVCL), as previously reported [10]. More specifically, a solution of 2 wt% of alginic acid was mixed with a mixture of NVCL monomer dissolved in a small amount of dimethylformamide (DMF), approximately 14 mmol, and a good homogenization was ensured. The polymerization reaction was performed at 75 °C for 5 h, mixing continuously. Purification of the copolymer from the free unbonded monomer was performed by precipitating the resulting solution in cold ethanol. The obtained product was purified via dialysis against twice-distilled water for one week to remove unreacted reagents (3.5 kDa cut-off dialysis membrane, Sigma-Aldrich, Hamburg, Germany), and the twice-distilled water was changed at least twice a day to maintain the ion concentration difference, and the final purified product was dried via lyophilization. After purification and lyophilization, the yield of copolymer synthesis has been determined gravimetrically as being 45%.

For the hydrogel preparation, the copolymer was dissolved in twice-distilled water (0.1 wt.%), and then Rifampicin (0.5 wt.%, with respect to the polymer’s matrix) with 2 mL of methanol was added to the final solution, under stirring (300 rpm, room temperature) for 6 h in order to obtain a homogenous mixture. The resulting solutions were frozen at −20 °C for 20 h and thawed at 25 °C for 4 h, and these procedures were repeated 3 times. The resulting porous hydrogel was maintained at 4 °C for other further characterizations. Freeze–thaw hydrogel with no drug was prepared using the same procedures.

### 3.3. Hydrogel Characterization

#### 3.3.1. FTIR and SEM Data

FTIR spectra were recorded on dried samples in KBr pellets using a Bruker Vertex 70 spectrophotometer (Berlin, Germany) and scanned within the range 400–4000 cm^−1^ in transmittance mode. Gold-coated cross-sections of hydrogels were examined with an SEM Tescan-Vega microscope (Brno, Czech Republic) at ambient temperature, with an operating voltage of 30 kV under vacuum conditions, and the morphology images were analyzed with ImageJ software v1.51.

#### 3.3.2. In Vitro Bioadhesion Tests

To evaluate the scaffold’s ability to connect with biological tissues, the bioadhesion tests were performed on a TA.XT Plus ^®^texture analyzer (from Stable Micro Systems, Godalming, UK) on a simulating biological membrane (cellulose membrane from a dialysis tubing 12 kDa, pre-boiled and cooled at room temperature, cut into 2 × 2 cm pieces, and maintained in PBS solution) [62]. The compressed hydrogels (pure and loaded with drugs, circular, ϕ = 8 mm) were fixed to the bottom of the graphite cylinder (P/8, with a double-glued thin membrane) and attached to the mobile arm. The cellulose membrane was placed on the holding device, and the physiological environment was simulated by moistening with 200 µL of phosphate-buffered solution (pH 7.2, 0.01 M) and immersion in a controlled temperature system (distilled water, heated at 37 °C, under stirring, 200 rpm). The mobile arm was lowered with a pre-determined speed of 1 mm/s and maintained in contact with the cellulose membrane for 30 s at a contact force of 9.80665 mN. The force–time plots were analyzed using Texture Exponent software ver. 6.1.18.0, and the maximum detachment force and the work of adhesion were calculated. Five replicates were measured.

#### 3.3.3. Drug-Loading Capacity and Release

To quantify the Rifampicin loaded in the hydrogel, an HPLC method was developed using a Shimadzu Nexera LC-40-XR system (Shimadzu, Kyoto, Japan) equipped with a serial dual plunger pump, an autosampler (SIL 40 XR), an SPD-40V series UV–Vis, and an RF-20Axs fluorescence detector. Chromatographic separation of Rifampicin was performed on a C18 column (2.1 × 150 mm, Waters CORTECS 2.7 μm), using two mobile phases: A (water/formic acid—99.9/0.1, *v*/*v*) and B (acetonitrile). Before use, the solvents were filtered through a 0.22 μm filter and degassed by ultrasonication. The injection sample was 10 μL, the run time was 10 min at isocratic mode (0.8 mL/min), and the optimal mobile phase ratio was A (60%): B (40%). The column temperature was kept at 30 °C during chromatographic operation with UV–Vis detection for Rifampicin (257 nm).

The quantitative analysis of Rifampicin was carried out based on the retention times and peak areas, respectively. LabSolutionDB software (Version 6.106SP1) was used for peak integration.

To plot the calibration curve, a stock solution (containing 2000 ppm) of standard Rifampicin was prepared by dissolving of 50.0 mg of Rifampicin in 25 mL MeOH 50%. From the stock solution, serial dilutions (0.5, 1.5, 2.5, 5, 10, 20, 40, 60, 80, and 100 ppm Rifampicin) were prepared. The experiments were performed in triplicate.

The calibration curve is a plot of the area under the peak (AU) to the external standard as a function of the drug concentration: AU = Slope × Concentration + Intercept

The slope and the intercept are determined from AU and concentration of Rifampicin. Using this equation, the Rifampicin from hydrogel was quantified.

In vitro Rif release model: The release study was performed using a phosphate buffer (pH 7.41). An amount of 0.0433 g of bare hydrogel and 0.0253 g of drug-loaded hydrogel was mixed with 50 mL phosphate buffer (pH 7.4) using a dialysis membrane. The mixture was then shaken at 80 rpm at 37 °C, and 500 μL of sample was collected at 1, 2, 4, 6, 8, and 24 h. The supernatant was filtered through a 0.22 μm filter, and then 10 μL of the sample was injected using HPLC method conditions. The experiment was performed in triplicate. The concentration (%) of Rif released from the hydrogel formulation into simulated fluids was calculated using the following equation:

$$RIf\;released\;\left(\%\right)=\frac{C_{1}}{C_{0}}\times 100\%,$$

where *C*_1_ = the concentration of Rif released in the simulated fluid, and *C*_0_ = the concentration of Rif loaded into the hydrogel.

#### 3.3.4. Antimicrobial Activity

In order to evaluate the antimicrobial activity, 4 bacterial strains (*Staphylococcus aureus*, MRSA (methicillin-resistant *Staphylococcus aureus*), *Escherichia coli*, and *Pseudomonas aeruginosa*) were cultured and tested. *S. aureus*, MRSA, *E. coli*, and *P. aeruginosa* are bacterial strains with a high potential to produce infections that are difficult to treat, as evidenced by the *mecA gene* of *S. aureus* and extended-spectrum β-lactamase (ESBL)-producing *E. coli*. Also, *P. aeruginosa* is known to have natural resistance to many antibiotics.

All these bacterial strains are reported yearly to *The European Centre for Disease Prevention and Control (ECDC)* from each European country. In Romania, in 2022, there were reported 22% isolates of *S. aureus* and 50% of *P. aeruginosa* from ICU (intensive care units) departments. From the bloodstream, in the case of septicemia, there have been reported *MRSA* and third-generation cephalosporin-resistant *Escherichia coli* in proportions of 48% and 71%, respectively [63]. These representative data, which underline the burden of MDR (multi-drug resistant) strains in our country, are analyzed by the *European Antimicrobial Resistance Surveillance Network (EARS-Net)*, the largest publicly funded system for antimicrobial resistance (AMR) surveillance in Europe, for each EU country. The objectives of *EARS-Net* are the implementation and improvement in national AMR surveillance programs and the improvement in diagnostic accuracy [63]. In this study, we have tested the drug-loaded hydrogel against the most frequent and MDR bacterial strains, with the hope of obtaining efficient results of infected wounds with these bacterial strains.

The pure and fresh culture (18 h) of each of the 4 bacterial strains were tested against rifampicin-loaded hydrogel (H-Alg-PNVCL-Rif), rifampicin pure substance (Rif) and unloaded hydrogel (H-Alg-PNVCL), following the Kirby–Bauer disk-diffusion protocol (108 CFU/mL bacterial suspension, homogenous inoculation of Mueller–Hinton blood agar) [64]. The tested antimicrobial substances were prepared to respect the classical antibiotic discs, which are 6 mm in diameter. The diameter of the inhibition area was measured in mm in transmitted light after 24 h and 48 h of incubation at 35 °C.

#### 3.3.5. In Vitro Cytocompatibility

##### MTT Assay

In vitro cytocompatibility tests were performed on normal human dermal fibroblasts (NHDF cell line, primary adult cells, Catalog Number C0135C GIBCO™, Thermo Fisher Scientific, Waltham, MA, USA). The cells were seeded in a 48-well culture plate at a density of 14 × 10^3^ cells/well for 48 h, until confluence, in cell-surviving environmental conditions (95% relative humidity 5% CO_2_, 37 °C, in DMEM-HAM F_12_ (DMEM supplemented with 10% FBS and 1% P/S/N)). The freeze-dried hydrogels were sterilized by exposure to UV (each side, 30 min) and then immersed for 24 h in DMEM-HAM F12. Samples were cut with a sterile biopsy circular scalpel and were included in 48-well plates with cells, and the cell culture progress was compared with control (cells with no hydrogels).

To evaluate the cell viability at 24, 48, and 72 h of culturing, the medium was replaced with MTT working solution (prepared as a solution, 0.25 mg/mL, in DMEM) and incubated at 37 °C for a period of time of 3 h. Finally, 500 μL DMSO was added to each well, and absorbance was measured at 570 nm (a Tecan Sunrise Plate Reader, Männedorf, Switzerland, was used). By adding DMSO, the resulting formazan (purple color) was solubilized, and the absorbance was correctly measured. The cell viability was calculated with the following relation:

$$\mathrm{Cell}\mathrm{viability}(\%)=\frac{abs\;sample}{abs\;control}\times 100,$$

where *abs sample* represents the absorbance of the solution from the well with hydrogel, while *abs control* represents the absorbance of the control (cells without hydrogel or drug). Each result represents the mean viability ± standard deviation (SD) of three independent experiments.

The cytotoxicity of Rifampicin was also evaluated. Rifampicin solutions with different concentrations (1 mg/mL, 5 mg/mL, and 10 mg/mL) were prepared and filtered before they were added to culture plates.

##### Cell Imaging

Live/dead staining assays were performed (Calcein-AM and May–Grünwald–Giemsa assays) in order to image and analyze the cells. In the Calcein-AM assay, the dye solution (1 µM/mL, in HBSS) was included in each well and incubated with cell cultures for 30 min at 37 °C, and the cells were imaged. In the May–Grünwald–Giemsa protocol, the cells were fixed with 3.7% formaldehyde for 24 h at room temperature. After that, the cells were washed with 1 × PBS (twice) and incubated with May–Grünwald dye (10 min at room temperature) and then with Giemsa dye for 25 min at room temperature; finally, the cells were washed with 1 × PBS (twice) and dried at room temperature. Images were taken in phase contrast using a Leica DM IL LED Inverted Microscope with a Phase-Contrast System (Leica Microsystems GmbH, Wetzlar, Germany) and compared with control cell cultures.

#### 3.3.6. Wound Healing Assay: Scratching Tests

The cell migration analysis demonstrates the wound-healing effect of hydrogels, and few protocols have been developed and reported [65,66,67,68]; in our experiments, some modifications were added to the reported methods. Normal human fibroblasts (NHDF cell line) were used to test the in vitro regenerative effect of hydrogels and the role of Rifampicin. The cells were seeded in the same conditions as in cytotoxicity tests (48-well culture plate at a density of 14 × 10^3^ cells/well, for 48 h, with DMEM-HAM F_12_ complete medium, at 37 °C in a 5% CO_2_ atmosphere to allow the cells to adhere to the well surface). After 48 h, a hollow was practiced in a vertical direction, and cells were detached using a 200 μL pipette tip. The remaining confluent cell monolayers were washed twice with HBSS (without Ca^2+^ and Mg^2+^) in order to remove the detached cells. DMEM-HAM F_12_ was added to each well, and then microscopic images of the area were taken to mark the time zero of the experiment. Sterilized and balanced hydrogels and filtered solutions of Rifampicin were added to the scratched layers of cells. The cell migration process was followed at different periods of time, and the viable cells were stained with the Calcein AM reagent. The dye solution, with a concentration of 2 µL/mL, was introduced into the cell cultures and allowed to undergo incubation for 30 min at a temperature of 37 °C and the images were obtained using a 10× objective lens from the Leica DM IL LED Inverted Microscope equipped with a fluorescence modulus (Leica Microsystems GmbH, Wetzlar, Germany). Experiments were performed in triplicate with similar results.

### 3.4. Statistical Analysis

Statistical analysis was performed by applying one-way ANOVA and Tukey post hoc analysis. Differences between groups were considered statistically significant for *p* < 0.05. All results were expressed as the mean of at least three experiments ± SD.

## 4. Conclusions

Hydrogel dressings based on sodium alginate-grafted poly(N-vinylcaprolactam) were successfully developed, incorporating Rifampicin as an antimicrobial drug through repetitive freeze–thawing cycles. The structural characteristics of the hydrogel and the polymer-drug compatibility were analyzed using FTIR, and interactions between alginate and Rifampicin were identified. Analysis through HPLC and spectrophotometry confirmed a concentration of 0.69% Rifampicin in the polymeric matrix. SEM analysis identified hydrogels with a porous structure, with varying sizes and shapes of pores, ranging from almost spherical geometries with smooth surfaces and high interconnectedness to lower porosity, non-uniform distribution, and irregular shapes in the presence of the drug. Both pure Rifampicin/unloaded hydrogel and Rifampicin-loaded hydrogel demonstrated antimicrobial activity against *S. aureus* and MRSA, with increased effects in the presence of the drug. The bioadhesive nature of the hydrogels, as evidenced by the force of detachment and work of adhesion, makes such compositions promising for wound dressings; the bioadhesive strength increased when Rifampicin was included in the matrix. The prepared hydrogels are cytocompatible and stimulate cell proliferation and mutual cell communication, and the in vitro scratch assay indicated that the formulated alginate-grafted poly(N-vinylcaprolactam) hydrogel with a small quantity of Rifampicin stimulates cell migration and wound closure. The obtained results suggest that the formulated matrix could be used as a delivery system to reduce the toxic effects of Rifampicin and is well-suited for wound healing and care applications.

## Figures and Tables

**Table 1:** Pore size variations for different hydrogel compositions.

Hydrogel	Min (µm)	Max (µm)
**H-Alg-PNVCL**	11.53 ± 7.15	77.05 ± 6.12
**H-Alg-PNVCL-Rif**	11.21 ± 7.18	331.32 ± 11.74

**Table 2:** The diameters of inhibition areas for H-Alg-PNVCL and H-Alg-PNVCL-Rif hydrogels and Rifampicin.

Bacterial Strains	Diameters of Inhibition Areas (mm)					
	H-Alg-PNVCL-Rif		Rif		H-Alg-PNVCL	
	24 h	48 h	24 h	48 h	24 h	48 h
*S. aureus*	26	28	40	42	16	18
MRSA	24	26	40	42	14	16
*E. coli*	no effect	no effect	25	27	no effect	no effect
*P. aeruginosa*	no effect	no effect	36	38	no effect	no effect

## Data Availability

The data presented in this study are available on request from the corresponding authors.

## Supplementary Materials

The following supporting information can be downloaded at https://www.mdpi.com/article/10.3390/ph17070943/s1, Figure S1. In vitro evaluation of NHDF migration in presence of different concentrations of Rifampicin (NHDF cell line, stained with Calcein AM at 4H, 6H and 8H); Figure S2. In vitro evaluation of NHDF migration in presence of H-Alg-PNVCL hydrogel and H-Alg-PNVCL-Rif hydrogel (NHDF cell line, stained with Calcein AM at 4H, 6H and 8H); Table S1. Optical images of Mueller Hinton plates for the tested hydrogels and drug.
