# Supplementary material for: In Vitro Biological Evaluation of an Alginate-Based Hydrogel Loaded with Rifampicin for Wound Care
Source: Pharmaceuticals (Basel). 2024 Jul 14;17(7):943. doi: 10.3390/ph17070943 (PMC11280071; doi:10.3390/ph17070943)
Supplement: Supplementary file 1 [file pharmaceuticals-17-00943-s001.zip › pharmaceuticals-3072874-supplementary.pdf]

In Vitro Biological Evaluation of an Alginate-Based Hydrogel Loaded with Rifampicin for Wound Care

Tudor Bibire 1,2, Radu Dănilă 2,3, Cătălina Natalia Yilmaz 4,* , Liliana Verestiuc 5,* , Isabella Nacu 5,6 , Ramona Gabriela Ursu 7 and Cristina Mihaela Ghiciuc 8,9
1 Doctoral School, Grigore T. Popa University of Medicine and Pharmacy, 16 Universitatii Street, 700116 Iasi, Romania; tudor_cd_bibire@d.umfiasi.ro
2 St. Spiridon County Clinical Emergency Hospital, 1 Independentei Blvd., 700111 Iasi, Romania; radu.danila@umfiasi.ro
3 Department of Surgery, Faculty of Medicine, Grigore T. Popa University of Medicine and Pharmacy, 16 Universitatii Street, 700116 Iasi, Romania
4 Biochemistry Division, Department of Chemistry, Faculty of Science, Dokuz Eylül University, Kültür Mah. Cumhuriyet Bulv. No:144 Alsancak, 35210 Izmir, Turkey
5 Department of Biomedical Sciences, Faculty of Medical Bioengineering, Grigore T. Popa University of Medicine and Pharmacy, 16 Universitatii Street, 700116 Iasi, Romania; cobzariu.isabella@gmail.com
6 Petru Poni Institute of Macromolecular Chemistry, 41-A Grigore Ghica Voda Alley, 700487 Iasi, Romania
7 Department of Microbiology, Faculty of Medicine, Grigore T. Popa University of Medicine and Pharmacy, 16 Universitatii Street, 700116 Iasi, Romania; ramona.ursu@umfiasi.ro
8 Department of Pharmacology, Faculty of Medicine, Clinical Pharmacology and Algeziology, Grigore T. Popa University of Medicine and Pharmacy, 16 Universitatii Street, 700116 Iasi, Romania; cristina.ghiciuc@umfiasi.ro
9 St. Maria Clinical Emergency Hospital for Children, 62 Vasile Lupu Street, 700309 Iasi, Romania
* Correspondence: duncaty@gmail.com (C.N.Y.); liliana.verestiuc@umfiasi.ro (L.V.)

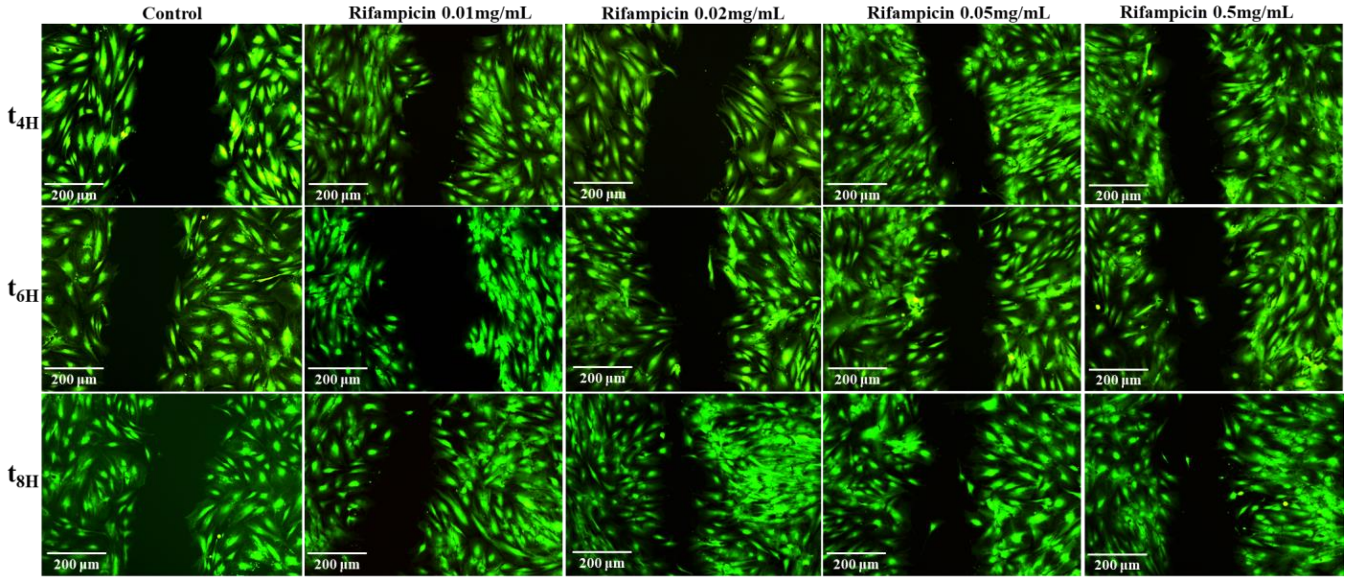

Figure S1. *In vitro* evaluation of NHDF migration in presence of different concentrations of Rifampicin (NHDF cell line, stained with Calcein AM at 4H, 6H and 8H).

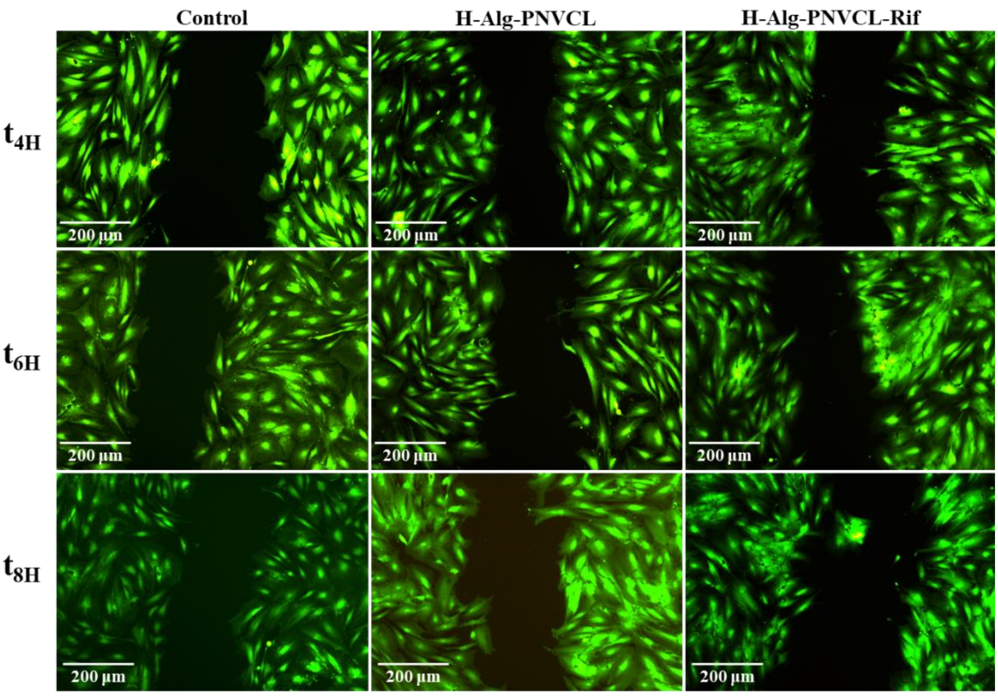

Figure S2. *In vitro* evaluation of NHDF migration in presence of H-Alg-PNVCL hydrogel and H-Alg-PNVCL-Rif hydrogel (NHDF cell line, stained with Calcein AM at 4H, 6H and 8H).

Table S1. Optical images of Mueller Hinton plates for the tested hydrogels and drug

Tested material/drug	*Staphylococcus aureus*	*MRSA*	*E coli*	*P. aeruginosa*
**H-Alg-PNVCL-Rif**	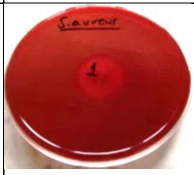	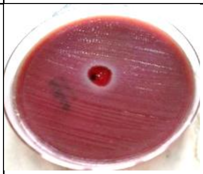	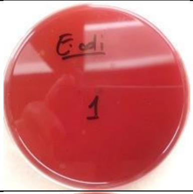	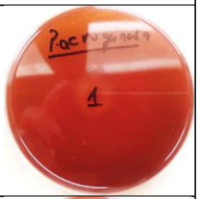
**H-Alg-PNVCL**	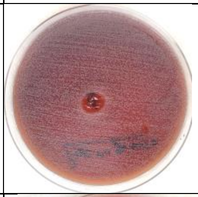	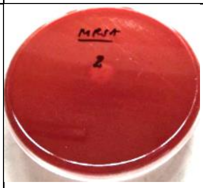	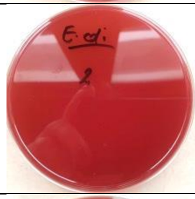	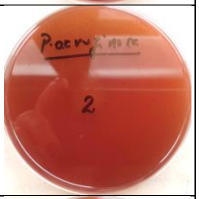
**Rif**	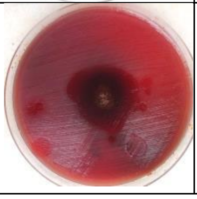	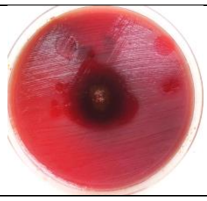	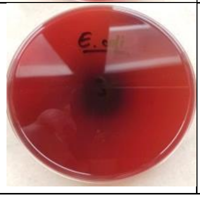	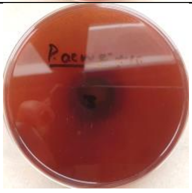
